# Expanded Polystyrene-Debris-Induced Genotoxic Effect in Littoral Organisms

**DOI:** 10.3390/toxics11090781

**Published:** 2023-09-14

**Authors:** Victor Pavlovich Chelomin, Nadezda Vladimirovna Dovzhenko, Valentina Vladimirovna Slobodskova, Andrey Alexandrovich Mazur, Sergey Petrovich Kukla, Avianna Fayazovna Zhukovskaya

**Affiliations:** Far Eastern Branch, V.I.l’ichev Pacific Oceanological Institute, Russian Academy of Sciences, Vladivostok 690041, Russia; chelomin@poi.dvo.ru (V.P.C.); doreme_07@mail.ru (N.V.D.); slobodskova@list.ru (V.V.S.); mazur.aa@poi.dvo.ru (A.A.M.); avianna@poi.dvo.ru (A.F.Z.)

**Keywords:** expanded polystyrene, oxidative stress, DNA damage, marine mollusks

## Abstract

Expanded polystyrene (EPS) is a major component of plastic debris in the environment, including coastal and littoral zones. EPS is widely used in various industries including fish farming and aquaculture, which poses a serious potential threat not only to cultured hydrobionts but also to all living organisms, including humans. This paper presents the results of experimental studies on the effects of EPS (0.024 m^2^/L) on marine mollusks *Mytilus trossulus* and *Tegula rustica*, which are typical inhabitants of the upper littoral of Peter the Great Bay (Sea of Japan), belonging to different systematic groups and differing in the type of nutrition. The results of biochemical marker analysis showed the development of oxidative stress processes. Thus, increasing malondialdehyde content relative to control values was registered in the digestive glands of *M. trossulus* and *T. rustica*. In the cells of the digestive glands of *M. trossulus,* integral antioxidant activity decreased more than 1.5 times compared with that of the control. The change in the concentration of protein carbonyls was unchanged in *M. trossulus*, whereas in *T. rustica,* there was a 1.5-fold increase. EPS exposure also resulted in significant DNA damage in the studied mollusks—the damage level increased 2.5-fold in *M. trossulus* and 1.5-fold in *T. rustica* relative to the control, indicating the genotoxic potential of EPS litters.

## 1. Introduction

The wide production of synthetic polymers has led to a rapid increase in plastic waste, polluting the environment. Waste plastic is disposed of, overflowing multiple disposal sites, carried by the wind and various water bodies, and, eventually and inevitably, into the marine environment. In the marine environment, synthetic polymers exposed to ultraviolet light, temperature, oxygen, a series of chemical transformations, and constant mixing lose their original properties, become fragile, and degrade into small fragments of varying sizes. As size decreases, plastic particles become more bioavailable and potentially more dangerous to various aquatic organisms [1,2]. This threat is now considered more of a priority than investigating the toxicological risks posed by the concentration of plastic litter, represented by large fragments of plastic products, on the seashore, littoral zone, and estuarine waters [3,4,5].

The littoral zone is a transit point for huge masses of plastic debris carried from the continent to the ocean. During their stay in the littoral zone, plastic fragments are concentrated and remain in close contact with a variety of marine organisms, mainly invertebrates, for an indefinite period.

In addition to the potential physical threat, plastic products and their fragments pose a chemical threat, as they are a complex mixture of chemicals of exogenous and endogenous origin. Numerous studies have shown that synthetic polymers can sorb a variety of pollutants from the environment on their surface and serve as a carrier from the source to the transit zone and then to the ocean [6,7,8,9].

Moreover, during polymer synthesis, various chemicals such as plasticizers, stabilizers, antioxidants, flame retardants, etc., are used as additives to improve their consumer properties [10]. Most of these adsorbed (exogenous) and “intrinsic” (endogenous) low-molecular-weight chemical compounds are bound to polymer structures physically rather than chemically and can be easily released into the environment [10,11,12,13,14,15,16,17,18,19]. Fluctuations in the major environmental factors (temperature, salinity, oxygen concentration, and UV) encourage the release of chemicals from synthetic polymers into the environment more easily. Therefore, it is the coastal zone where plastic debris mainly accumulates that experiences its dangerous toxic effects [5,19,20,21,22].

Given the significant accumulation of plastic debris on shores and in the littoral zone of marginal seas, the leaching of unreacted monomers and chemical additives from large fragments of plastic can seriously affect the total level of pollution in this transit zone. The ecological implications of these processes are clear, as various micro and macro forms of marine organisms, including their planktonic stages, are exposed to the risk of indirect chemical exposure through desorption and diffusion into the marine environment. In addition, the transport of released chemical compounds from plastic fragments through the water column to littoral organisms is an important food chain transmission pathway and a threat to human health.

Although multiple studies have confirmed the leaching of a wide variety of toxic chemicals from different types of plastic under laboratory conditions [2,11,17,23], the real situation in the littoral zone necessitates the study of the response of littoral organisms living in this environment to the effects of a range of chemicals leached from plastic.

Expanded polystyrene (EPS) shows a range of unique physical properties (lightness, stiffness, relative strength, and thermal and electrical insulation) and is one of the most widely used polymers in the world. This polymer, with a global production of more than 20 million tons per year, is widely used in a variety of global industries, including textiles, electronic device housings, food packaging, soft toys, building insulation materials, and a variety of marine aquaculture and fisheries products [15,24,25,26,27].

Among the EPS products, disposable items are the most common, which quickly become waste, where they are dispersed and fragmented in the environment. EPS fragments have now become a major component of plastic debris, forming huge accumulations along coastlines and in the littoral zone of marginal seas [15,19,28,29]. This is particularly apparent in a number of regions with high population density and developed industry, i.e., the East Pacific coast of Asia. Researchers from South Korea [19,30] have shown that marine plastic debris is dominated by various fragments of polystyrene foam, especially polystyrene foam buoys, which are widely used in fisheries and numerous aquaculture farms. In South Korea alone, these industries use about 2 million of such buoys annually, of which no more than 28% are sent for recycling [4].

The aim of our study was to clarify the ecotoxicological risks for marine organisms living in the littoral zone in the presence of high volumes of plastic debris based on the response of biochemical markers of oxidative stress. To evaluate these concerns, we conducted a series of ecotoxicological experiments involving EPS macrofragments and two littoral mollusks (*Mytilus trossulus* and *Tegula rustica*) differing in their mode of nutrient uptake. *M. trossulus* is a bivalve filter feeder mollusk capable of absorbing not only dissolved organic matter from the water but also organo-mineral particles corresponding to the size of phytoplankton cells. This experimental approach has been used previously by Li et al. [31]. *T. rustica* is a typical representative of gastropod mollusks, which are characterized by grazing activity—scraping fine food particles and phytobenthos from the surface of soil [32,33,34].

## 2. Materials and Methods

### 2.1. Experiments Design

The study was carried out at the marine experimental station “Popov Island” of the V.I. Il’ichev Pacific Oceanological Institute. Mollusks *M. trossulus* and *T. rustica* were sampled in Alekseev Bay (Sea of Japan) by SCUBA divers. After acclimatization (5 days) in laboratory conditions at a stable temperature (17 ± 0.5 °C), constant aeration, and daily water change, the mollusks were placed in 20 L tanks at a rate of 0.9 L per individual. All walls of the experimental tanks, including the lid, were lined with fragments of EPS (0.024 m^2^/L). During the experiment (3 d), water in the tanks was not changed, a stable temperature was maintained (17 ± 0.5 °C), and animals were not fed additionally. In total, there were 6 tanks: 3 control, 3 experimental (*n* = 3), in each of which there were 10 specimens of *M. trossulus* and 10 specimens of *T. rustica*.

After 3 d, the digestive gland was extracted from the mollusks to determine the index of the total antiradical activity and measure the concentration of malondialdehyde and protein carbonyls. For this purpose, the tissues were homogenized in a homogenizer (SilentCrusher S; Heidolph Instruments, Schwabach, Germany) in a 0.1 M phosphate buffer, pH = 7.5. Freshly extracted tissue was used to assess DNA damage.

### 2.2. Integral Antioxidant Activity

The integral antioxidant activity (IAA) of the tissue homogenates was determined from the suppression of the oxidation reaction of 2,2′-azinobis (3-ethyl-benzothiazoline-6-sulfonic acid) (ABTS) by the peroxyl and alkoxyl radicals formed during the thermal decomposition of 2,2′-azobis (2-methyl-propionamidine) dihydrochloride (ABAP) [35]. The reaction mixture was prepared in a phosphate buffer (0.1 M) and incubated at 37 °C. The measurements were made using a Shimadzu UV-2550 spectrophotometer with a thermostatted cell at a wavelength of 414 nm. The magnitude of activity was calculated with a calibration plot using 6-hydroxy-2,5,7,8-tetramethylchloraman-2-carboxylic acid (Trolox) (Sigma Aldrich, Taufkirchen, Germany).

### 2.3. Malondialdehyde

The level of malondialdehyde (MDA) was calculated by the formation of a colored reaction during the interaction of the tissue homogenate with 2-thiobarbituric acid, as described in [36]. To prevent lipid peroxidation during the determination of MDA, an alcoholic solution of butylhydroxytoluene (Merck KGaA, Darmstadt, Germany. CAS-no 128-37-0) was added to the samples to a final concentration of 5 mM. The content of malondialdehyde was determined via the color reaction with 2-thiobarbituric acid (TBA, Merck KGaA, Darmstadt, Germany. CAS-no 504-17-6).

Then, 30% trichloroacetic acid (Germany, AppliChem GmbH Ottoweg 4 D-64291 Darmstadt. CAS-no 76-03-9) and a 0.75% TBA solution were added sequentially to the tissue homogenate. The mixture was thoroughly mixed and heated for 20 min in a water bath (Memmert WNB 7, Memmert GmbH + Co. KG, Aeussere Rittersbacher Strasse 38 D-91126 Schwabach) at a temperature of 95 °C. After cooling, sediments were separated from the samples by centrifugation at 1500× *g* for 20 min. The measurements were carried out at wavelengths of 580 nm and 532 nm, then the difference in the readings of the optical density was found. To calculate the MDA content, the molar extinction coefficient (1.56 × 10^5^ cm^−1^/M^−1^) was used. The measurements were carried out on a Shimadzu UV-2550 spectrophotometer.

### 2.4. Protein Carbonyls

For the determination of protein carbonyls, the digestive gland was homogenized in 0.05 M phosphate buffer (pH 7.0) supplemented with 1 mM phenylmethanesulfonyl fluoride (PMSF) to inhibit proteases. Carbonyl groups of proteins in the digestive gland and gills were determined via the alkaline method [37]. In total, 400 µL of DNPH (10 mM in 0.5 M H_3_PO_4_) was added to 400 µL of the protein solution followed by 10 min of incubation in the dark. Then, 200 µL of NaOH (6 M) was added. The time exposure for this mixture was 10 min as well. Absorbance was read at 450 nm on a Shimadzu UV-2550 spectrophotometer. The concentration of carbonyl groups in the investigated solutions was calculated using the molar absorptivity of 22,000 cm^−1^M^−1^. The Lowry method was used to determine the protein concentration [38]. The Lowry method is based on two reactions. The first one consists of the formation of a complex of copper cations (Cu^2+^) with the amide bonds of the protein, followed by the reduction of copper under alkaline conditions. The resulting product is called a biuret chromophore, which is stabilized by the addition of tartrate. In the second reaction, the resulting copper–protein complex is reduced with Folin’s reagent. In this case, the protein solution turns blue. The transparency of the solution is determined spectrophotometrically at a wavelength of 660 nm. Calibration curves were built using solutions of bovine serum albumin, the concentrations of which were calculated based on the molar extinction coefficient.

### 2.5. DNA Damage

To determine the level of damage in the DNA, we used an alkaline version of the comet assay adapted for hydrobionts [39].

The digestive glands were gently extracted from the mollusks and washed several times with 4 °C isotonic solution (500 mM NaCl, 12.5 mM KCl, 5 mM EDTA-Na2, and 20 mM Tris-HCl, pH 7.4). After washing and removing mucus, the digestive glands were carefully cut with scissors in 4–5 mL of CMFS buffer. The cell suspension was filtered from large tissue fragments through a sieve with a cell diameter of 40 mm. The cells in the filtrate were precipitated via centrifugation and resuspended in an isotonic solution at a concentration of 10^5^ cells/mL. Next, after 10–20 min of incubation, the isolated cells were used in the comet assay.

Then, 50 µL of the cell suspensions was added to 100 µL of 1% fusible agarose (MP Biomedicals, Eschwege, Germany) in 0.04 M phosphate buffer (pH 7.4) at 37 °C, resuspended, and then placed on a slide previously coated with 1% agarose solution and covered with a coverslip. The sample was placed in a refrigerator for 3 min to solidify the agarose.

After the removal of the coverslip, the slide was transferred to a lysis solution (2.5 M NaCl; 0.1 M EDTA-Na2; 1% Triton X-100; 10% DMSO; 0.02 M Tris, pH 10) for 1 h in a place protected from light at 4 °C. At the end of the lysis, the slides were washed with distilled water at a temperature of 4 °C and placed in an electrophoresis buffer (300 mM NaOH; 1 mM EDTA-Na2) and incubated for 40 min. Electrophoresis was performed at 2 V/cm for 15 min, followed by a neutralization step (0.4 M Tris-HCl, pH 7.4). Slides were stained with SYBR Green before imaging.

To assess the extent of DNA damage, we used an Axio Imager A1 fluorescence microscope (Carl Zeiss) with an AxioCam MRc digital camera to digitally image DNA comets. For each sample, an image of at least one hundred DNA comets was maintained. Then, using the computer program CASP (version V 1.2.2.; CASPLab, Wroclaw, Poland. https://casplab.com, accessed on 5 August 2023), the comets captured were processed. The level of cell DNA damage was assessed through determining the parameter characterizing the proportion of DNA in the comet tail.

### 2.6. Fourier-Transform Infrared Spectroscopy

FTIR spectra were acquired using an IRAffinity-1S (Shimadzu, Japan) equipped with attachment frustrated total internal reflection (wavenumber range of 4000–400 cm^−1^, 32 scans per spectrum, spectral resolution of 4 cm^−1^). The background was measured with the same settings against air. The obtained spectra were processed using LabSolutions IR software (Shimadzu, Japan).

### 2.7. Statistical Analysis

The data collected during all experiments were processed using MS Excel and Statistica 10 software packages. The assumptions of normality and homogeneity were assessed using Levene’s and Shapiro–Wilk’s tests, respectively. Data on the DNA content in the tail of the comet and the biochemical markers did not achieve normality, and nonparametric tests of variance Kruskal–Wallis ANOVA followed by pair-wise Mann–Whitney tests were conducted. Results were considered statistically significant when *p* < 0.05.

## 3. Results

### 3.1. Integral Antioxidant Activity

**Figure 1:**
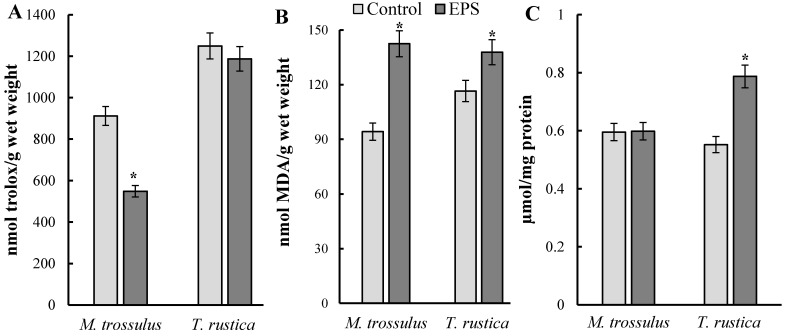
Changes in (**A**) integral antioxidant activity, (**B**) malondialdehyde, (**C**) protein carbonyls in digestive glands of mollusks *M. trossulus* and *T. rustica* exposed to expanded polystyrene (mean ± standard deviation, *n* = 3). *—difference from control is significant at *p* ˂ 0.05.

After exposure to EPS in *T. rustica*, no significant differences were found in the level of IAA between the control and experimental groups. However, in cells of the digestive gland of *M. trossulus*, this index decreased more than 1.5 times compared with that of the control (*p* = 0.034), which indicated a decrease in the activity of the low-molecular link of the antiradical system and in the pronounced toxic effect with the development of oxidative stress in the organism (Figure 1A).

### 3.2. Malondialdehyde

Exposure to ESP increased the MDA concentration in the digestive gland of *M. trossulus* and *T. rustica* (Figure 1B). In the digestive gland of *M. trossulus,* the MDA level was 142.5 µmol/g wet weight, which differed from that of the control (84.2 µmol/g wet weight) by more than 1.5 times (*p* = 0.032). In *T. rustica*, the increase in the MDA concentration was slightly lower and was 1.2-fold (*p* = 0.034). The data obtained indicate that the mollusks exposed to EPS were in a state of significant oxidative stress.

### 3.3. Protein Carbonyls

EPS exposure did not increase the concentration of protein oxidized forms in the digestive tissue of *M. trossulus* (Figure 1C). However, in *T. rustica*, this parameter increased significantly compared with that of the control by almost 1.5 times (*p* = 0.016).

### 3.4. Comet Assay

To evaluate DNA damage in the digestive gland cells of control and experimental mollusks of both species, the comet assay method was applied, which allows estimating this parameter in individual cells.

**Figure 2:**
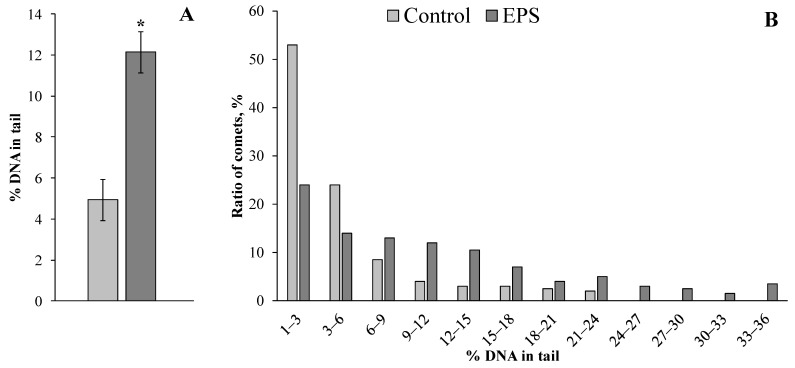
Changes in levels of DNA damage in digestive glands of *M. trossulus* exposed to expanded polystyrene (mean ± standard deviation, *n* = 3). *—difference from control is significant at *p* ˂ 0.05.

**Figure 3:**
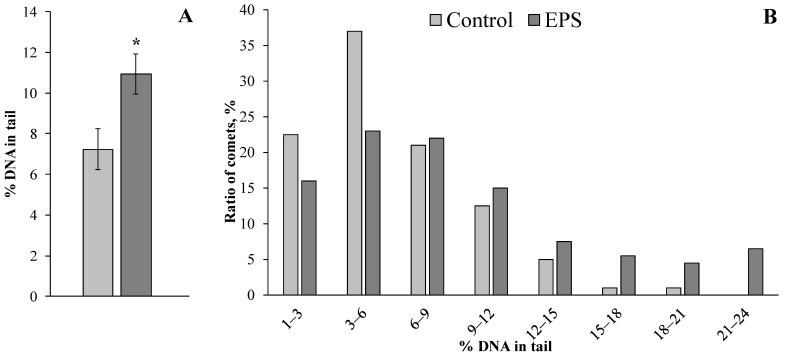
Changes in levels of DNA damage in digestive glands of *T. rustica* exposed to expanded polystyrene (mean ± standard deviation, *n* = 3). *—difference from control is significant at *p* ˂ 0.05.

Exposure of the mussels *M. trossulus* and the gastropods *T. rustica* to EPS resulted in an increase in the DNA damage level in the cells of the digestive gland by almost 2.5 (*p* = 0.034) and 1.5 (*p* = 0.034) times compared with that of control mollusks, respectively (Figure 2A and Figure 3A). For a more detailed analysis, the comets that were formed from the digestive gland cells’ DNA in control and experimental mollusks of both species were grouped according to the level of genome damage (with a 3% range) and are presented in Figure 2B and Figure 3B.

Almost 98% of the cells of the digestive glands of the control groups of mussels had a level of nuclear DNA damage not exceeding 20%. In the experimental mollusks after EPS exposure, not only the proportion (<82%) of such cells with a relatively low level of genome damage decreased dramatically but also comets with more than 30% of DNA migrating to the “tail” were recorded (Figure 2B). A similar pattern was observed in experimental gastropods after exposure to EPS. Only in experimental mollusks were cells (about 10%) with DNA damage levels exceeding 20% recorded (Figure 3B).

## 4. Discussion

High concentrations of plastic debris, mainly EPS, in the narrow littoral zone may impact littoral organisms. These concerns are valid given that synthetic polymers, including EPS, are chemical complexes in which chemical additives are weakly bound to the matrix and can leach into the surrounding environment [15,16,40,41]. Moreover, in this zone, due to the high variation in physical and hydrochemical factors, the primary processes of polymer degradation, fragmentation, and transformation are active, accelerating the leaching of chemicals [28,42,43,44,45].

It is practically impossible to repeat under laboratory conditions all factors of such an unstable zone influencing the leaching of chemical additives. Therefore, in our experiments, we only roughly simulated real conditions by maintaining temperature, oxygen saturation, and water turbulence during the experiments.

Although our experiments were short-term, the results showed that EPS exposure resulted in changes at the biochemical level in the digestive glands of both mollusk species. Considering the specific characteristics of the biochemical markers used, there is evidence to suggest that oxidative stress processes with increased genome destruction were initiated in the tissues of the experimental animals to varying degrees. From the analysis of the above data (Figure 1), it follows that these negative processes were more pronounced in the experimental mussels *M. trossulus* than in the gastropod mollusk *T. rustica*. Thus, in EPS-exposed *M. trossulus*, the cells of the digestive gland demonstrated significantly decreased IAA and increased content of the end product of oxidative destruction of lipids—MDA. However, the increase in pro-oxidative processes did not impact the protein cell components. A different picture was found in the experimental gastropod *T. rustica*. Against insignificant changes in IAA level and a low significant increase in the content of lipid peroxidation products, a significant accumulation of oxidized proteins was observed. Apparently, the differences in the response of oxidative stress biomarkers to EPS exposure are due to the specifics of the physiology of dissolved nutrient uptake and the state of the antioxidant system in these species.

The concentrations of oxidized forms of lipids and carbonyl groups of proteins in experiments on marine invertebrates are not always directly related. This may be the result of a process referred to in the literature as feedback inhibition [46]. In which the formation of lipid peroxidation acts as an antioxidant mechanism preventing the formation of protein carbonyls through the formation of adducts between peroxides and proteins while inhibiting proteolysis.

Another possible explanation for the differences between lipid peroxidation and carbonyl formation is the fact that different oxidizing agents produce different products. So Dalle-Don et al. [47] showed that there are substances capable of causing the formation of carbonyls with a slight formation of lipid peroxidation and a slight oxidation of the DNA molecule; metals are also capable of primarily causing the oxidation of proteins and a number of oxygen radicals that lipids attack first.

The results are in agreement with the literature data reported in the study of other plastic types. Different types of polymers interacting with marine organisms induce various negative changes at the molecular and biochemical levels, associated with the generation of reactive oxygen species (ROS) and the development of oxidative stress processes [1,48,49,50,51,52,53].

The comet assay results indicated that EPS caused an increased destabilization of the DNA molecule structure integrity of the digestive gland cells in both species of experimental mollusks (Figure 2 and Figure 3). The comet assay is a sensitive method that allows the assessment of the changes in the nucleus of individual cells at early stages of genome destruction. Considering the importance of genome integrity in the functioning of biological systems of different levels of organization, note that DNA damage is also prognostic, indicating potential risks for the development of distant negative effects. Therefore, the increased level of DNA damage in the cells of the experimental mollusks revealed in our experiments should be attributed to the most important effects of EPS toxicity.

Although the specific mechanism of ROS generation upon inert particles of synthetic polymer exposure is still unclear, a few experimental studies in recent years have linked the genotoxic properties of polymers to an increased generation of ROS [1,2,54,55,56,57,58]. We may assume that EPS presence in the environment of experimental mollusks affects the activity of biochemical systems involved in the processes of ROS generation. This results in an imbalance between pro- and antioxidant systems at this stage, which causes increased genome fragmentation and the generation and accumulation of lipid (MDA) and protein (PC) oxidative degradation products.

We did not analyze the seawater to identify the chemical constituents leaching from EPS during the experiments. However, based on the design of our model experiments and the responses of the biochemical markers, it is reasonable to assume that a chemical factor was the leading factor behind the pro-oxidant biochemical changes in both mollusk species. Furthermore, this view is based on multiple studies indicating that EPS contains several chemical components that are relatively easily and rapidly leached into the environment [13,18,26,27,59,60,61]. More than 40 low-molecular-weight chemicals were detected in EPS [27], as well as ethylbenzene [59], bisphenol A (BPA) [61], and toxic metals (Hg, Pb) [62,63]. However, from an ecotoxicological point of view, the main components of EPS, such as styrene and its oligomers [16,64] and hexabromocyclododecane (HBCD) [15,18,28,65,66], pose serious risks to marine organisms.

Styrene and its oligomers, products of incomplete polymerization, are hazard group 2A (carcinogenic risk) and are classified as endocrine-disrupting chemicals [58]. Due to the ease with which they migrate from EPS objects into the environment, styrene and its oligomers are widely detected in coastal waters around the world and serve as indicators of this polymer’s presence in the environment [16,64].

Hexabromocyclododecane is the main chemical additive (0.7–2.5%) used for the fire safety of EPS products [61]. This brominated antiperene is not covalently bound to the polymer and thus can be easily released from EPS fragments into the environment. This is supported by studies [13], which showed the transfer of HBCD from EPS to the mussel *Mytilis edulis*. The accumulation of HBCD in the earthworms *Eisenia fetida* and *Metaphire guillelmi* has been noted when polystyrene foam microfragments are ingested with food [65]. In the hepatopancreas of the shrimp *Litapenaeus vannamei*, this antiperene induced a range of important biochemical changes and initiated oxidative stress, expressed as a significant accumulation of MDA [66]. Due to its toxic properties and tendency to bioaccumulate, HBCD was included in Annex A of the Stockholm Convention on Persistent Organic Pollutants [18].

**Figure 4:**
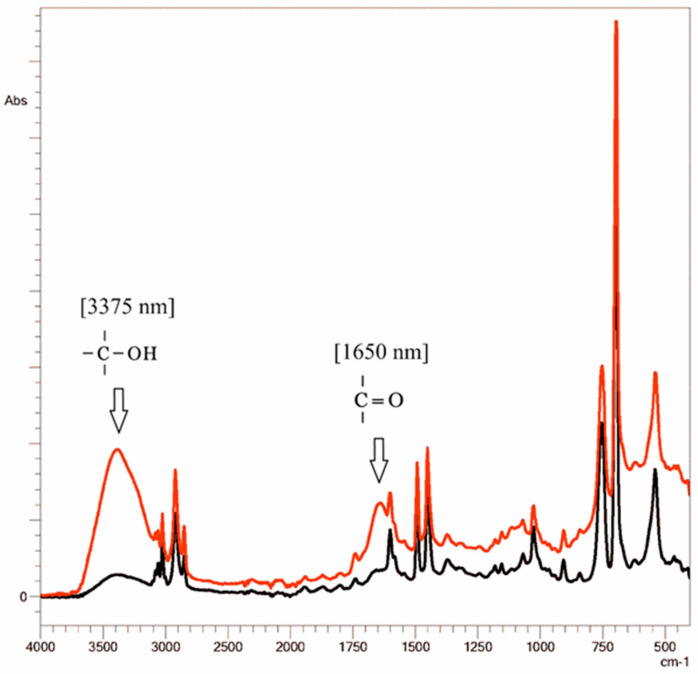
FTIR spectra of EPS before (black line) and after (red line) the experiment.

Expanded polystyrene is an unstable polymer that rapidly (within 2–4 days) degrades and fragments under conditions close to real conditions in the environment [26,45]. The FTIR spectroscopy spectra of EPS fragments before and after the experiments were compared (Figure 4) and severe chemical changes in the surface layer of the polymer were found. Particularly significant changes were detected in the absorption bands in the regions of 1600–1700 cm^−1^ and 3200–3600 cm^−1^, corresponding to carbonyl and hydroxyl groups. These changes indicate the occurrence of oxidative processes in the EPS surface layer [42,43,45], which not only increase the leaching of chemical additives from the polymer [28,42] but also encourage the formation of toxic water-soluble products [41,67,68].

Therefore, a special study by Thaysen et al. [59] showed that a complex of chemicals leached from EPS used in food and drink packaging showed toxicity by reducing the survival and reproductive capacity in the daphnia *Ceriodaphnia dubia*. Given the potential risks of EPS products in households, some municipalities in South Korea have even banned the use of disposable EPS products.

Our results show that synthetic polymers such as EPS pose a real hazard not only through uptake by biota but also indirectly through the water column into which various highly toxic chemicals migrate from the polymer. Apparently, the biochemical changes initiated by EPS are more extensive and are not limited to the oxidative stress and genotoxicity identified in our work. Thus, EPS products released into the marine environment should be considered as an additional source of marine pollution by chemicals toxic to marine invertebrates of the littoral zone.

## Data Availability

Not applicable.
